# Modelling Catalyst Surfaces Using DFT Cluster Calculations

**DOI:** 10.3390/ijms10104310

**Published:** 2009-11-20

**Authors:** Izabela Czekaj, Jörg Wambach, Oliver Kröcher

**Affiliations:** Energy Department, Paul Scherrer Institute, 5232 Villigen PSI, Switzerland; E-Mails: joerg.wambach@psi.ch (J.W.); oliver.kroecher@psi.ch (O.K.)

**Keywords:** DFT, cluster model, *in situ* DRIFTS, *in situ* XPS, metal-support interactions, reaction mechanism

## Abstract

We review our recent theoretical DFT cluster studies of a variety of industrially relevant catalysts such as TiO_2_, γ-Al_2_O_3_, V_2_O_5_-WO_3_-TiO_2_ and Ni/Al_2_O_3_. Aspects of the metal oxide surface structure and the stability and structure of metal clusters on the support are discussed as well as the reactivity of surfaces, including their behaviour upon poisoning. It is exemplarily demonstrated how such theoretical considerations can be combined with DRIFT and XPS results from experimental studies.

## 1. Introduction

One of the long-term targets of research in heterogeneous catalysis is to gain an understanding of the processes of interest on a molecular level, in order to discover general catalysis principles and/or to develop better catalysts based on rational design. With this goal we started a few years ago to investigate: (i) detailed surface-reaction mechanisms, and (ii), surface modifications during catalytic reactions (processes like oxidation, reduction, clustering, deactivation, and so on). For a given reaction, we study both commercial catalysts and home-made model catalysts with identical or at least similar composition, and follow the surface processes and modifications applying a complimentary combination of surface science analytical techniques (*e.g.,* XPS, DRIFTS) under *in situ* or *quasi in situ* conditions [1]. However, a comprehensive view of the processes occurring on the catalyst surface can only be obtained by combining these results with modelling of the reaction mechanism using *ab initio* DFT methods [2,3]. In our present studies we use a cluster model, which is often applied in modelling of catalytically active centres, for geometrical representation of the surfaces [4,5].

Our activities are part of the projects of the General Energy Research Department of the Paul Scherrer Institute (PSI). Within the broad research portfolio covered at PSI, we have focused our interest on a sustainable energy supply for future mobility by catalytic conversion of renewable primary fuels (*e.g.,* biomass) to secondary fuels (hydrogen, methane) via synthesis gas (CO + x H_2_) and on the urea-SCR processes for the removal of NO_x_ from diesel vehicles. One example to be shown will illustrate how the production of methane from reformer gas induces surface modifications on a Ni/Al_2_O_3_ catalyst [1]. The influence of biomass derived synthesis gas on the surface properties of the catalyst and on its reactivity under methanation conditions was investigated by combined High Pressure Reaction Cell (HPC)–XPS experiments. Recently, theoretical studies were performed to understand the molecular structure of the Ni/Al_2_O_3_ catalyst and why nickel particles detach from the surface during methanation.

Another example which we will discuss is the investigation of catalysts for the urea-SCR process. A variety of different catalysts can be used for SCR processes, such as TiO_2_ or Al_2_O_3_ for the urea decomposition and more complex systems, such as V_2_O_5_/WO_3_-TiO_2_ or metal-exchanged zeolites, for the actual SCR reaction. We will exemplarily show the differences in the reaction mechanisms of the hydrolysis of isocyanic acid (HNCO) on anatase TiO_2_ (101) and γ-Al_2_O_3_ [6–8], which were revealed combining *ab-initio* DFT calculations using a cluster model with *in situ* DRIFTS investigations. Furthermore, a characterization study on the deactivation of V_2_O_5_/WO_3_-TiO_2_ SCR catalysts [9] by alkali metals originating from additives or impurities from fuels and lubrication oils has been performed. Combination of theoretical calculations (excited state of core electrons) and experimental XPS studies has been used for describing the role of alkali metals in blocking the catalytically active sites.

## 2. Computational Details

The calculations described in this paper were performed with clusters models. We used density functional theory (DFT) methods (StoBe program code [10]) together with the non-local generalized gradient corrected functionals (RPBE) according to Perdew, Burke, and Ernzerhof [11,12]. All Kohn-Sham orbitals are represented by linear combinations of atomic orbitals (LCAOs) using extended basis sets of contracted Gaussians from atom optimizations [13,14]. Detailed analyses of the electronic structure in the clusters are carried out using Mulliken populations [15] and Mayer bond order indices [16,17]. The calculations of the vibrational frequencies were performed with harmonic approximations as well as an anharmonicity fit in the Morse potential function, as implemented in the StoBe code [18]. Full geometry optimization of the adsorbed species and the active centers on the clusters were performed. Atoms were allowed to move in the 3-dimentional space without constraints until equilibrium has been reached. Double zeta valence polarization (DZVP) type was used for the orbital basis sets, which we found to be sufficiently accurate (see [7]). Theoretical vibrational spectra were obtained by convolution of the vibrational spectra of the individual adsorbates, applying Gaussian line-shapes. The frequencies are reported as obtained from the calculations, without scaling.

In our studies the catalyst surfaces were modelled by clusters of different size and geometry, which were saturated by hydrogen atoms with R_OH_ = 0.97Å. For modelling the TiO_2_ (101) surface, Ti_2_O_9_H_10_, Ti_8_O_28_H_24_, Ti_13_O_43_H_34_ and Ti_15_O_50_H_40_ were selected as clusters [6], among which the Ti_8_O_28_H_24_ cluster proved to be best suited for further reaction studies. In case of the γ-Al_2_O_3_(100) surface, Al_11_O_30_H_27_, Al_15_O_40_H_35_ and Al_25_O_58_H_41_ clusters were studied, from which Al_11_O_30_H_27_ was finally selected for adsorption and Al_15_O_40_H_35_ for Ni cluster deposition study. Three different clusters of nickel were deposited at γ-Al_2_O_3_ surface (Ni_2_, Ni_7_, Ni_9_) [2,3]. In case of V_2_O_5_ (010) surface, the V_6_O_20_H_10_ cluster has been used for surface representation [9].

## 3. Results and Discussion

### 3.1. Modelling of Metal-Support Interactions

Our first example for the combination of DFT calculation with experimental studies of heterogeneous catalysts deals with the production of methane from synthesis gas. The effect of the composition of synthesis gas, starting with pure CO + H_2_ and approaching in stepwise fashion the composition as delivered from an existing wood gasifier, on the surface properties of a commercial Ni/Al_2_O_3_ catalyst and on its activity under methanation conditions was studied on an atomic level by *quasi in-situ* X-ray photoelectron spectroscopy (XPS) [1]. One of the conclusions was that the stability of the Ni particles on the γ-Al_2_O_3_ support can be influenced by cluster growth phenomena, which influence both size and distribution of the metal particles.

**Figure 1.**
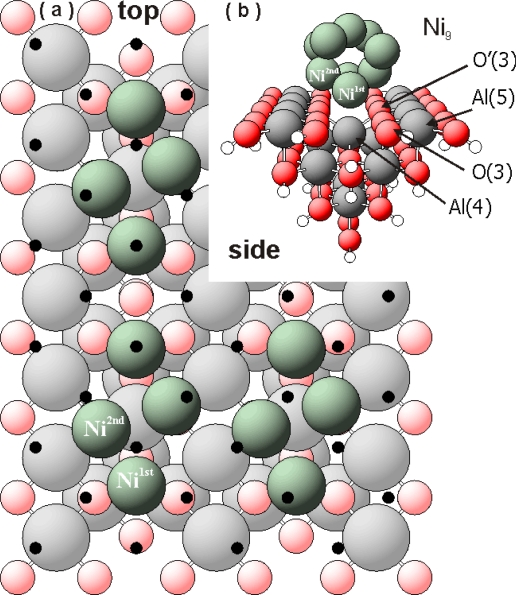
Ni/Al_2_O_3_ cluster; colour code: (Ni: grey spheres; Oxygen: red spheres; Hydrogen: white spheres). (a) Topography (top view) of the interfacing Ni atoms (green spheres) for the relaxed Ni_9_ cluster on an Al_15_O_40_H_35_ cluster; all Ni interface atoms included (θ_Ni,interface_∼0.4 mL). Black dots symbolize the position of Ni atoms of an ideal Ni(100) monolayer; (b) side view onto a relaxed entire Ni_9_ cluster (θ_Ni_∼0.4 mL) bound on an Al_15_O_40_H_35_ cluster.

In order to shed light on the involved metal-support interactions, a theoretical study has been performed [2,3]. It was shown that the deposition of a very low number (Θ_Ni_ < 0.4 mL) of metal atoms on γ-Al_2_O_3_ only influences the local surface structure, in particular the neighbouring centres of Ni, such as: Al(4), Al(5), O(3) and O’(3) (see previous paper [3]). The stabilization energy of deposited nickel particles on Al_15_O_40_H_3_*_5_* cluster was calculated as difference between the total energy of the metal deposited on the γ-Al_2_O_3_ surface and sum of the total energies of pure Al_15_O_40_H_3_*_5_* and the metal atoms, respectively. In all cases, the first layer of nickel on Al_2_O_3_(100) deposits in positions closer to octahedral O(3) centres (see Table 1) with different stabilization energies per Ni atom (for Ni_2_: −0.82 eV, for Ni_7_: −0.90 eV and for Ni_9_: −0.71 eV). The calculated stabilization energies for the different Ni clusters are comparable with published energies obtained for the adsorption of different metals, mainly Pd, on γ-Al_2_O_3_ [19,20]. Being strongly bound to O(3) centres, the deposited nickel influences the electronic structure of the γ-Al_2_O_3_, too. The largest investigated nickel cluster (Ni_9_) creates many interesting structures at the support surface. Figure 1a shows only the interface atoms (green spheres) of the Ni_9_ cluster after relaxation. The interfacing Ni atoms correspond to a coverage θ _Ni_∼0.40 mL. The figure was created by multiplying the calculated Ni/Al_2_O_3_ cluster in x and y direction (see Figure 1b). Black dots symbolize the mismatch of an “ideal” Ni(100) monolayer with the γ-Al_2_O_3_ surface. Strong vertical and lateral re-arrangements of the interface Ni atoms in the Ni_9_ clusters with respect to the position on the Ni(100) surface are indicated by our DFT results. Part of the interfacing nickel atoms (Ni^1st^) seem to prefer the “valley regions” between the AlO_5_ rows, close to the O(3) centres. These O(3) centres are mostly influenced creating strong bonds with the first (interfacing) nickel atoms.

**Figure 2.**
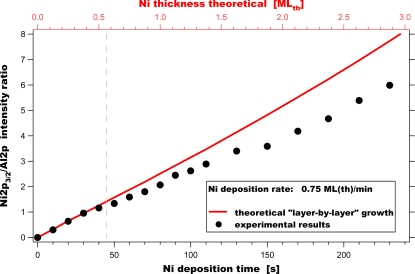
Changes of Ni 2p_3/2_/Al 2p ratio during deposition of nickel onto the Al_2_O_3_ support. The red line corresponds to the theoretical expectation of development of the Ni 2p_3/2_/Al 2p ratio for a layer-by-layer growth. The dashed line indicates the proposed start of the 3D cluster growth.

This finding is in good accordance with our experimental results (see Figure 2), where we observed the existence of electronically strongly altered Ni adsorbed on γ-Al_2_O_3_ in the first stage of the deposition. Our experimental studies suggest that Ni does not form clusters immediately, but one-dimensional (or possibly small two-dimensional) agglomerates, initially. This seems to be valid for nickel coverages up to Θ_Ni_∼0.5 mL. Further growth of these initial agglomerates following a layer-by-layer growth mode (see the red line) was not found. Our data clearly show a deviation from the expected behaviour exhibiting smaller Ni 2p_3/2_/Al 2p values. This indicates that in the second stage three-dimensional Ni clusters are formed on the surface. We conclude that Ni deposition on γ-Al_2_O_3_ follows a “modified” Stranski-Krastanov growth mode under the applied experimental conditions, which is in accordance with the findings of Jacobs *et al*. [21].

Summarizing, we conclude that nickel is stabilized on the γ-Al_2_O_3_ surface influencing the electronic properties of the newly formed surface. Our DFT data suggest that at low coverages (≤0.2 mL) Ni prefers being localized in AlO_4_ tetrahedra between rows of AlO_5_. The DFT results are in good agreement with the experimentally obtained results from the initial stage of Ni deposition, where the formation of a “partial Ni monolayer” is suggested. Further Ni deposition first leads to three-dimensional agglomerates, which are finally transferred to Ni clusters on the surface by continued Ni deposition, as was derived from the slow approach of the XPS binding energy to the value of bulk Ni. For Ni_7_ and Ni_9_ clusters, the initially deposited Ni atoms, which represent the interface nickel atoms (“Ni^1st^”), are bound strongly to the oxygen of the support and are located in positions closer to O(3) centres as well as between rows of AlO_5_ with adsorption energy, which varies with the size of the cluster. The astonishingly good agreement between the experimental data and our theoretical studies shows the good accuracy of cluster model calculations for investigating metal-support structures.

### 3.2. Water Adsorption on Metal Oxide Surfaces: TiO_2_ and Al_2_O_3_

The effects of water adsorption on different catalysts are of high importance, because water is present in many processes, such as the methanation of syngas or the SCR process with humid exhaust gas. TiO_2_ and Al_2_O_3_ are interesting materials for both processes, which are discussed as urea decomposition catalysts or as support for methanation catalysts.

**Figure 3.**
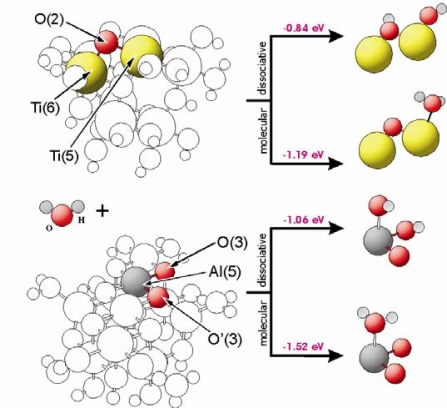
Geometric structure of water at TiO_2_ and Al_2_O_3_. Colour code: (oxygen: red, titanium–yellow, aluminium–dark gray, hydrogen–light gray).

Molecular and dissociative adsorption of water is possible on both substrates [22–28]. Figure 3 shows the corresponding geometric and the electronic structures of the surfaces for both types of catalysts. The calculations of the water adsorption showed that the M(5) centres (M = Ti, Al) are involved on the TiO_2_ as well as the Al_2_O_3_ surface. Concerning dissociative adsorption, one hydrogen atom of the water molecule is transferred to and stabilized at a surface oxygen centre. In case of TiO_2_, O(2) centres are able to bind the hydrogen atom. For Al_2_O_3_, the situation is more complicated, because at the surface exist two types of 3-fold coordinated oxygen centres, O(3) and O’(3). The O(3) centres are bound to Al(5) and Al(6) centres, while O’(3) are linked to Al(5) and Al(4) centres. As visible in Table 1, the oxygen in O(3) has a lower charge and it is stronger bound than in case of the O’(3) centres. Our results show that hydrogen prefers to be adsorbed on the O(3) oxygen sides. In case of hydrogen bound to O’(3) centres, our optimisation results indicate an immediate hydrogen transfer to the O(3) centres. Water can be stabilized molecularly at a M(5) centre with an adsorption energy of −1.19 eV and −1.52 eV for TiO_2_ and Al_2_O_3_, respectively, whereas water adsorbs dissociatively with an adsorption energy of E_ad_ = −0.84 eV and −1.06 eV on both materials at a M(5) centers.

Table 2 shows the calculated characteristic vibrational frequencies of water being bound to TiO_2_ and Al_2_O_3_. Although molecular adsorption of water should be energetically even more favoured, dissociative adsorption of water is observed over the whole temperature range and even at low temperatures (50 °C/323 K) [29].

**Figure 4.**
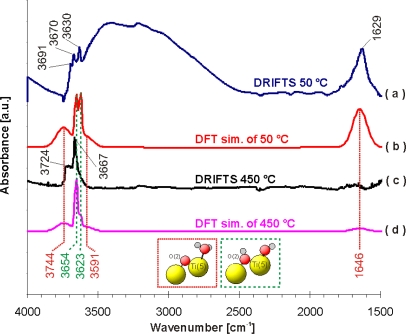
Theoretical IR spectra and experimental DRIFT spectra of the adsorption reaction experiments dosing ∼200 ppm H_2_O onto fresh TiO_2_ at T = 50 °C and 450 °C. (a) DRIFT spectrum at 50 °C. (b) Theoretical IR spectrum at 50 °C. (c) DRIFT spectrum at 450 °C. (d) Theoretical IR spectrum at 450 °C. Vertical lines correspond to calculated wave numbers for (1) molecular adsorption of water at Ti_8_O_28_H_24_ (red dotted lines), and (2) dissociative adsorption of water at Ti_8_O_28_H_24_ (green dashed lines).

Figure 4 shows a comparison between simulated and measured IR spectra of water adsorbed on TiO_2_. Only bands deriving from water adsorption are visible in case of the theoretical spectrum. The band at 1,629 cm^−1^ found in the experimental DRIFT spectrum at 50 °C is in good agreement with the vibrational frequency of 1,646 cm^−1^ [δ(H-O-H)] obtained from DFT calculations for molecularly adsorbed water with respect to the used approximations. There is also a fair match between the experimental band at 3,691 cm^−1^ in the DRIFT spectrum and the theoretical band at 3,744 cm^−1^ [ν(O(2)H)] in the “DFT” spectrum. On first view, it seems that the experimental band at 3,630 cm^−1^ is also caused by molecularly adsorbed water. However, we believe that this band can rather be attributed to dissociatively adsorbed water, which was found to have a theoretical frequency of 3,623 cm^−1^ [ν(O(1)H)]. The experimental bands at 3,670/3,667 cm^−1^ in the DRIFT spectra at 50 °C and 450 °C, respectively, are also compatible with dissociatively adsorbed water with a calculated band at 3,654 cm^−1^ [ν(O_s_(2)H)] (see green dotted line in Figure 4). The stretching vibration at 3,623 cm-1 [ν(O(1)H)] is connected with hydroxyl groups adsorbed at the Ti(5) centres and the stretching vibration at 3,654 cm^−1^ [ν(O_s_(2)H)] results from hydroxyl groups formed with the O_s_(2) surface oxygen atoms [O_s_(2)-H]. However, a detailed analysis of the experimental spectra at 450 °C (see Figure 4c) shows that the absorbance of the ν[O(1)H] vibration is much weaker than that of ν[O_s_(2)-H] one, which suggests a higher population of O_s_(2)-H sites (with theoretical frequency 3,654 cm-1, see Figure 4d). A lower population of hydroxyl groups at Ti(5) centres is a very important feature for the adsorption of HNCO on these sites as a prerequisite for the hydrolysis reaction. It should be noted that the DRIFT spectrum of TiO_2_ at 50 °C shows also a broad band in the range 2,500–3,740 cm^−1^, which is typical for the presence of liquid water due to the formation of hydrogen bridging bonds [30].

The comparison of the calculated vibration frequencies with the DRIFT experiments suggests that at lower temperatures (50 °C) both molecular (bands at 1,629 and 3,691 cm^−1^) and dissociative (bands at 3,630 and 3,670 cm^−1^) adsorption of water occurs with higher amounts of molecularly adsorbed water. At higher temperatures (450 °C) mainly dissociative adsorption (bands at 3,667 and 3,724 cm^−1^) was observed (see Figures 4c and d).

The combination of theoretical and experimental vibrational spectroscopic studies allows the identification of surface species and how they are adsorbed on the surface and show in detail which species remain on the surface even after drying of the catalyst. We have demonstrated such a comparison for water adsorption, which always occur under humid process conditions on heterogeneous catalysts, but it is also useful for studying other adsorbates, for example isocyanic acid, which is hydrolyzed over TiO_2_ in the urea-SCR process.

### 3.3. Isocyanic Acid Behaviour at Different Catalysts

Isocyanic acid (HNCO) is formed from the thermolysis of urea, which is used as an ammonia precursor compound in the selective catalytic reduction of nitrogen oxides in diesel engines. HNCO itself hydrolyses to ammonia and carbon dioxide. The hydrolysis of isocyanic acid is possible over a variety of metal oxides, among which TiO_2_ and Al_2_O_3_ were selected to study the mechanism both theoretically and with *in situ* DRIFT investigations.

The first calculation has been made for the isocyanic acid molecule in the gas phase in order to validate the electronic parameters’ accuracy. A comparison between results from DFT codes (StoBe and Gaussian98) and experimentally derived vibrational frequencies of pure HNCO are shown in Table 3. Very good agreement of theoretical frequencies and experimental data of Ranier *et al*. [31] has been found for the calculations with the StoBe code.

**Figure 5.**
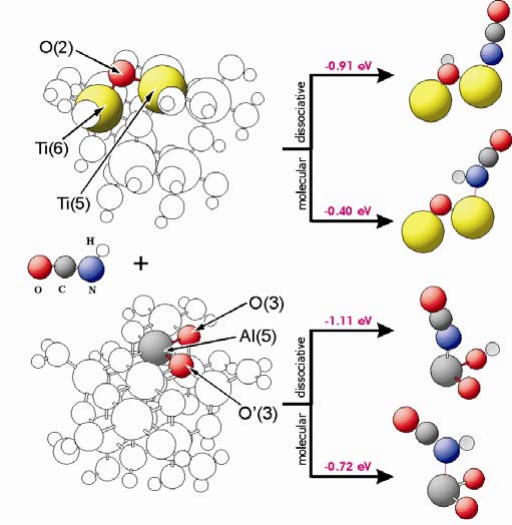
Geometric structure of isocyanic acid at TiO_2_ and Al_2_O_3_. Colour code: (oxygen–red, nitrogen–blue, carbon–black, hydrogen–light gray, titanium–yellow, aluminium–dark gray).

As a next step the adsorption of isocyanic acid TiO_2_ and Al_2_O_3_ has been investigated. The calculations revealed that dissociative as well as molecular adsorption of HNCO is possible and energetically feasible on both the TiO_2_(101) [6,7] and the Al_2_O_3_(100) surface [8,32,33]. Figure 5 shows adsorption energies as well as geometric structures of HNCO interacting with different sites of TiO_2_(101) and Al_2_O_3_(100). These surfaces are represented by Ti_8_O_28_H_24_ and Al_11_O_30_H_27_ clusters, respectively. The HNCO or –NCO groups are stabilized at the M(5) centres of these clusters. In case of dissociative adsorption on Al_2_O_3_, hydrogen is bound to the O(3) oxygen side in parallel to the adsorption of water (see paragraph 3.2). The HNCO molecule is bound to the M(5) centres with stabilization energies of −0.40 eV and −0.72 eV for TiO_2_ and Al_2_O_3_, respectively. The energies for dissociative adsorption are −0.91 eV and −1.11 eV for TiO_2_ and Al_2_O_3_, respectively. As shown in Figure 3, water adsorbs dissociatively with an adsorption energy of E_ad_ = −0.84 eV (TiO_2_) and −1.06 eV (Al_2_O_3_). This means that dissociative adsorption of isocyanic acid is favourable, and competitive with respect to water adsorption.

Due to the fact that the catalysts are working under humid conditions, any investigation of the reaction mechanism of the HNCO hydrolysis has to consider co-adsorption of HNCO and H_2_O on the catalyst surface. This approach comprises the competitive adsorption of water and isocyanic acid, as well as the interaction of water with isocyanic acid adsorbates. It also requires the presence of free neighboured five-fold coordinated metal sites.

**Figure 6.**
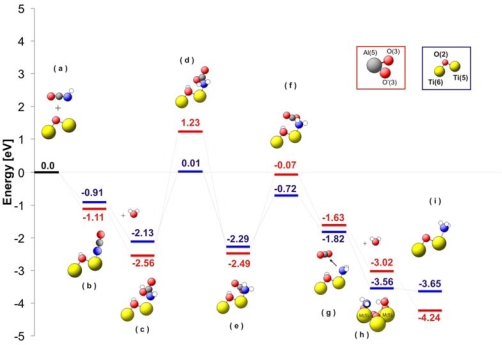
The energy diagram for the hydrolysis of HNCO on TiO_2_(101) (blue levels) and γ-Al_2_O_3_ (100) (red levels). Colour code: (oxygen–red, nitrogen–blue, carbon–black, hydrogen–light gray, titanium–yellow, aluminium–dark gray). At the picture only centers active in adsorption are shown. (a) Cluster + HNCO reference level. (b) Dissociative adsorption of HNCO on the catalyst. (c) Water attack on the NCO group at the M(5) site and formation of surface carbamic acid (NHCOOH). (d) Transfer of the carboxyl H to the NH group. (e) Formation of adsorbed carbamate (NH_2_CO_2_) at the M(5) site. (f) CO_2_ separation from the NH_2_ surface group. (g) CO_2_ desorption and stabilization of the NH_2_ group at M(5). (h) Migration of the H from adsorbed water to NH_2_. (i) NH_3_ at the M(5) site.

**Figure 7.**
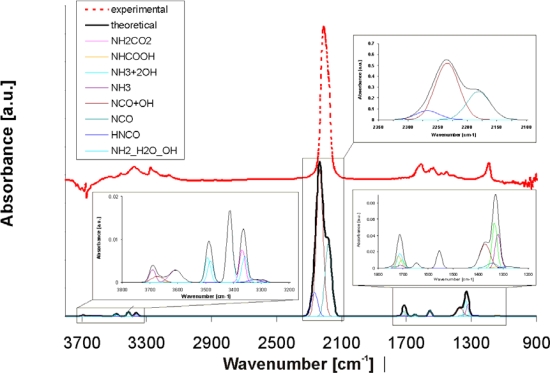
Theoretical vibrational IR spectra and experimental DRIFT spectrum of HNCO adsorption (70 ppm) on TiO_2_ at 150 °C (experimental procedure: 15 min HNCO adsorption followed by 15 min nitrogen purging). The insets show enlarged areas of certain areas of the theoretical spectrum and relate the origin of the peaks to the individual compounds (see colour code). For that list of vibrational frequencies (cm^−1^) of individual surface intermediates see also Table 4.

Figure 6 shows a comparison of the energy levels for the different intermediates observed during hydrolysis of isocyanic acid. The first important step of the reaction mechanism is dissociative adsorption of HNCO (see Figure 6b). After stabilization at the surface, –NCO groups are attacked by a water molecule. As consequence of this water attack, a carbamic acid complex is formed (-NHCOOH) without energy barrier. The carbamic acid is strongly adsorbed on the surface with an energy of −2.13 eV for TiO_2_ and −2.56eV for Al_2_O_3_. Thereupon, the carbamic acid changes its conformation in a highly endothermic process transferring hydrogen from carbonylic oxygen to the nitrogen atom (see Figure 6d). Consequently, a carbamate complex (–NH_2_CO_2_) is formed at the surface (Figure 6e). This carbamate complex decarboxylates (Figure 6g) and the NH_2_ group remains at the M(5) site. The formation of ammonia requires an additional hydrogen atom, which can be obtained from a water molecule adsorbed on a neighbouring M(5) site. This hydrogen transfer from a second water molecule is facilitated by the very low energy level of the system after the adsorption of water (−3.56 eV for TiO_2_ and −3.02 eV for Al_2_O_3_; Figure 6h). The NH_3_ is finally released from the catalyst surface enriching the catalyst surface with OH groups. This is in agreement with the *in situ* DRIFTS experiments described below, since strong OH/water vibrations are visible after reaction always. As an additional proof for the accuracy of our reaction mechanism a comparison of the theoretical vibrational spectra of the reaction intermediates of the HNCO adsorption on TiO_2_ and Al_2_O_3_ with the *in situ* DRIFT spectrum for TiO_2_ has been performed. An example of these spectra is shown in Figure 7 for the TiO_2_ catalyst.

In the following, we give a brief introduction how to obtain theoretical IR spectra. The calculations of the vibrational frequencies were performed using harmonic approximations with additional anharmonicity fit as discussed in Chapter 2 [18]. The vibrational spectrum of the individual adsorbates includes all vibrations and their characteristic intensities (see Table 4). The frequency of a vibration is based on mechanical properties (anharmonically oscillating atomic masses) whereas its intensity is a function of the change in dipole moment [34]. The amplitude of a peak in a vibrational spectrum is proportional to the square of the first derivative of the dipole momentum of the molecule with respect to one of the normal-mode vibrational coordinates (a combination of nuclear displacement coordinates of the equilibrium geometry).

Therefore in our case the intensity of a certain vibrational mode of an adsorbate is a function of the square of its dipole momentum. The final spectrum of an individual adsorbate has been described by Gaussian line shapes function with the same line width (50 cm^−1^) in order to obtain realistic peak shapes.

A complete theoretical vibrational spectrum was obtained by convolution of the vibrational spectra of all individual adsorbates determined to be present in the considered reaction path under the reaction condition applied (*e.g.,* water or hydroxyl groups adsorbed at neighboured metal centres), applying Gaussian line-shapes. The wavenumbers are reported as obtained from the calculations. The adsorption of isocyanic species with different surroundings has been considered with presence or absence of water, for example NCO and NCO_aq_, see Table 4. Due to the fact that in DRIFT spectroscopy the measured intensity of a certain vibration is a function of the number of adsorbed molecules, the theoretical spectrum of the individual adsorbates/intermediates have to be scaled separately to simulate the different populations of the individual adsorbates. The aim is to obtain the best possible fit to the experimental data.

The detailed theoretical spectrum comprising all individual adsorbates/intermediates, which are considered for the hydrolysis of isocyanic acid, is shown in Figure 7. The red curve shows the experimental DRIFT spectrum of the system HNCO/TiO_2_ without water at 150 °C. A strong band at 2,209 cm^−1^ together with less intense bands in the range 3,524–3,163 and 1,619–1,190 cm^−1^ are visible. The strong band at 2,209 cm^−1^ is assigned to the asymmetric stretching vibrations of –NCO groups adsorbed on the surface. From our theoretical studies we found that the main contribution to this band comes from dissociatively adsorbed isocyanic acid, stabilized on M(5) centres, evoking the vibration at 2,234 cm^−1^ in the neighbourhood of strong OH groups (see brown individual curve) and the vibration at 2,181 cm^−1^ in the absence of OH groups (light blue individual curve). However, small amounts of HNCO adsorbed in molecular form can be found at 2,266 cm^−1^ (light blue individual curve), which is very close to the vibration of gaseous HNCO (2,259 cm^−1^).

In the case of the Al_2_O_3_ catalyst the theoretically predicted vibrations of the -NCO groups are at 2,274 cm^−1^ in case of dissociative adsorption and at 2,264 cm^−1^ in case of molecularly adsorbed HNCO. These vibrations are also in good agreement with experimental data presented recently by Ozensoy *et al.* [22], who recorded TD-FTIR spectra at 120 °C and who found a band at 2,254 cm^−1^. The calculated vibrations of NCO_aq_ and HNCO are very close to each other suggesting that on Al_2_O_3_ rather isocyanic acid in molecular form can be found. This is in good agreement with the higher adsorption energy of molecular HNCO on Al_2_O_3_ (−0.72 eV) compared to TiO_2_ (−0.40 eV) (see Figure 5).

The theoretical bands below 1,800 cm^−1^ and above 3,000 cm^−1^ in Figure 7 have a similar shape as in the experimental TiO_2_ spectrum, but they are shifted to higher frequencies. This could be due to differences in mass, bond strength or other effects. The low frequency bands in the 1,200–1,800 cm^−1^ range in the calculated spectrum correspond to the bending vibrations of NH_2_ or NH_3_ as well as NH_2_CO_2_ and NHCOOH and the symmetric stretching vibrations of NCO influenced by water. The high frequency bands in the 3,000–3,500 cm^−1^ range are the sum of the N-H stretching vibrations of the individual adsorbates influenced by water.

The intermediate complexes, such as –NCO or carbamic acid, have higher stabilisation energies at the Al_2_O_3_ surface, which simply means that it will be more difficult to convert them into the following surface complexes. This finding is in agreement with our experimental result that Al_2_O_3_ has a lower activity than TiO_2_ for the HNCO hydrolysis.

### 3.4. Theoretical Modelling of Binding Energies of Different Elements

As the last example we have selected the investigation of the deactivation of V_2_O_5_/WO_3_-TiO_2_ SCR catalysts by lubrication oil additives, which is of high significance for the NO_x_ reduction in diesel vehicles. In this project the influence of dopant metals on the binding energies of oxygen in a V-W-Ti-O catalyst was investigated theoretically by modelling the doping of VO_x_ [9]. In this type of catalyst the active face are two dimensionally spreaded VO_x_ surface polymers and consequently, we limited our study on the influence of the metals on the active face only.

**Figure 8.**
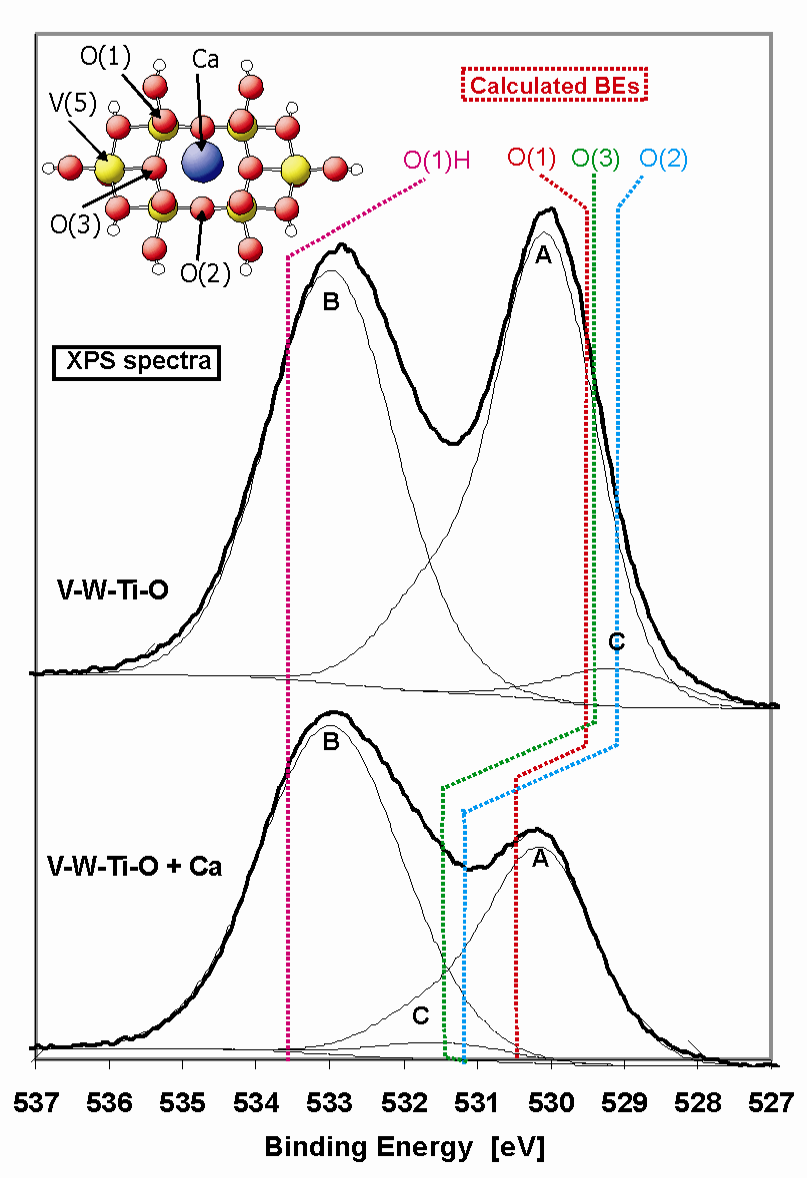
XP spectrum (Mg Kα) and deconvolution of the O 1s BE region for the pure (V-W-Ti-O) and Ca-poisoned (V-W-Ti-O + Ca) samples. Vertical lines refer to the calculated BE’s. In upper left corner the V_6_O_20_H_10_ cluster is shown with the characteristic active centres.

The two dimensional VO_x_ surface species were described by clusters, which consist of six vanadium atoms, V_6_O_20_H_10_, as shown as inset in Figure 8. The V_6_O_20_H_10_ cluster represents all possible single-, double- and three-fold coordinated oxygen centres and five-fold coordinated vanadium centres on a orthorhombic V_2_O_5_(010) surface as well as the particular hole between the vanadyl-oxygen centres, where dopant metals are especially stabilized. Two dopant metals were tested, K and Ca, which were positioned in the energetically favourable hole position between vanadyl oxygen centres, following theoretical studies of Witko *et al*. [36].

XPS analyses of the individual components of the V-W-Ti-O catalyst, mainly V_2_O_5_, TiO_2_, WO_3_ and TiO_2_-WO_3_, was carried out to obtain information about binding energies and full-width-at-half-maximum data for the oxygen O1s region. The spectra were measured in our VG ESCALAB 220i XL set-up using the Mg X-ray source applying 15 kV and 30 mA in large area geometry. For a more detailed description see ref. [9]. Sample charging was in the order of 10eV. The binding energy (BE) scale was adjusted by setting the main C1s peak to 284.5 eV. The spectra were deconvoluted applying Gaussian-Lorentzian line-shapes and a Shirley-type background.

As background for calculations of theoretical binding energies the Koopmans theorem can be taken. If the ground state of the system is considered and assuming the remaining electrons as being inert, then the binding energy of a particular electron is equal to the negative energy of the orbital eigenvalue: E_b_ = −ɛ_b_. However, especially valence electrons respond due to the removal of a photoelectron, and also their relaxation and correlation energy must be included in the binding energy calculations. The relaxation and correlation energy can be included by calculating the binding energy as difference of the total energy between the ground state system (E_tot_^Ground^) and the final state after emission of a particular electron, and the electron hole in the system (E_tot_^Final^):

$${\text{E}}_{\text{b}}=-{ɛ}_{\text{b}}-{\text{E}}_{\text{tot}}{\;}^{\text{Ground}}+{\text{E}}_{\text{tot}}{\;}^{\text{Final}}+{\text{E}}_{\text{corr}}$$

This definition is more accurate then Koopmans theorem and guarantees that relaxation of the remaining electrons is included.

XPS binding energies of the O 1s region of the V_6_O_20_H_10_ cluster were calculated as the total energy difference between ground state and the core ionized state [37]. The theoretically obtained binding energies were shifted by i) subtracting the sample work-function, and by ii) adding a relativistic correction (+0.33 eV for O) [38]. The effective core potentials (ECP) were used for all other oxygen atoms to localize the core hole on the particular oxygen centres, O(1), O(2) and O(3).

In Figure 8 the O 1s XP spectrum of the pure and metal-doped V-W-Ti-O catalyst is presented. The spectra exhibit a two-peak structure at about 530 eV and 533 eV, which can be correlated with a TiO_2_-WO_3_ (peak A) compound and hydroxyl groups (peak B), respectively. For the deconvolution of the measured spectra, reference samples of single and mixed metal oxides were used (TiO_2_, WO_3_, V_2_O_5_ and TiO_2_-WO_3_, see Table 5). Peak A (529.8 eV) and peak B (533.0 eV) were kept fixed during the fitting. The derived binding energies are listed in Table 5. The asymmetry in peak A is caused by oxygen in WO_3_ that has a higher binding energy (531.7 eV) and therefore TiO_2_-WO_3_ has been chosen as most suitable reference sample for this peak. Additionally, a small peak at about 529 eV can be distinguished (peak C, see Figure 8, V-W-Ti-O sample), which is likely connected with V_2_O_5_. During deconvolution the ratio between the content of V_2_O_5_ and TiO_2_ in the catalyst has been taken into account. In the upper spectrum of the V-W-Ti-O catalyst, the peak at lower BE corresponds quite well with our theoretical predictions of the binding energies of the different surface oxygen groups (see Table 5 and Figure 8) of pure V_2_O_5_. The binding energies of oxygen on a pure V_2_O_5_ (010) surface are localized at about 529.3 eV, which is exactly the position of peak C in case of the V-W-Ti-O sample.

The terminal oxygen, O(1), is most important for the SCR reaction [36], for which the possible formation of OH group was considered in the calculation of the oxygen binding energy in O(1). The binding energy of terminal oxygen of Brønsted acidic V-OH site is equal 533.5 eV, which corresponds well with the second prominent peak (BE about 533 eV). However, it has to be noted that Brønsted acidic sites could also come from other components of the catalyst, mainly TiO_2_.

Doping of the V-W-Ti-O with Ca leads to a decrease of the O1s signal located at the lower binding energy. Additionally, the peak labelled as “B” starts to be more asymmetric towards the lower binding energy range. We assign this asymmetry to the appearance of a third peak “C” at an energy position between 531–532 eV (see Figure 8, V-W-Ti-O + Ca), which is in agreement with a theoretically determined binding energy shift after Ca-doping (see Table 5). The theoretically determined binding energies of oxygen at the metal doped V_2_O_5_(010) surface are also shifted to higher energies (centred at 530.9 eV). However, the predicted shift of the oxygen binding energies was somewhat lower than the experimental value. A reason for this may be the additional shift of O binding energies in real V-W-Ti-O system compared to pure V_2_O_5_. However, the trend of the O1s peak shift is similar and offers a proper explanation of the role of the metal-dopant. Changes in the O1s signal after doping is connected with the formation of strong bonds between surface oxygen centres and the metal-dopant as well as an electron transfer from the dopant to the oxygen centre. Consequently, the oxygen becomes increasingly negatively charged and therefore exhibits a higher binding energy. Due to the charge transfer and a coupling with additional elements, the surface oxygen is supposed to be less reactive than in pure V-W-Ti-O catalyst.

## 4. Conclusions

After many years of DFT investigations, the level of theoretical insight into the electronic structures, geometries, stabilities and adsorption properties of model catalysts is now reaching a level that the investigation of more complex catalytical systems becomes possible with this method. In our research activities we try to build a bridge between applied industrial catalysts and model systems at an atomic level by using a combination of experimental and theoretical methods. Following this approach, the methodology for the investigation of catalytic systems has been discussed on the basis of the following examples: (i) investigations and modeling of metal-support interfaces, (ii) modeling of reaction mechanisms by comparison of theoretical and experimental DRIFT spectra, (iii) investigation of catalyst deactivation by combination of experimental and theoretical XPS spectra.

The shown examples clearly demonstrated that state-of-the-art cluster calculations are well suited for studying model as well industrially relevant catalysts, since always a good fit of theoretical calculations and experimental data (DRIFT, XPS spectra) was reached. Insight was provided into the catalyst structures and their functionality in the investigated reactions. In the first example, we could successfully describe the structure and deactivation mechanism of an industrial Ni/Al_2_O_3_ catalyst by a cluster model. Secondly, we resolved the reaction mechanism of the isocyanic acid hydrolysis over TiO_2_ and successfully interpreted the corresponding DRIFT spectra and, thirdly, cluster model calculations helped us to deconvolute XPS spectra and to understand deactivation phenomena of vanadia-SCR catalysts. The results obtained so far on the hydrolysis of isocyanic acid indicate that even a computational screening might be feasible for identification of better catalytic materials for this reaction.

In our opinion, cluster models are especially suited for the description and prediction of materials, in which small clusters are catalytically active. Another important advantage of cluster models is the possibility to study the interactions of adsorbates with an ideal catalyst surface as well as with single defects (*e.g.,* vacancies) at low surface coverages.

However, several challenges still remain on the long road of a complete understanding of real-world catalysts: (i) a more detailed explanation of imperfections, *e.g.,* defects, steps, and their effects on the reactivity of catalysts is required, (ii) a more detailed description of complex systems is needed, where different material are either the support or the supported phase, and (iii) it is necessary to improve the models for theoretical spectra of complex systems. It would be desirable to use both cluster and periodic cell models for the same problems in future and to compare the results in order better avoid errors coming from limitations of both approaches.

## Figures and Tables

**Table 1.** Results obtained by DFT calculations summarising atomic charges (eV), distances (Å) and bond orders (Mayer bond analysis) of (a) the pure Al_15_O_40_H_35_ cluster, (b) the Ni_9_/Al_2_O_3_ cluster.

**Cluster:**	Al_15_O_40_H_35_	Ni_9_/Al_15_O_40_H_35_
**Centre**	**Charge [eV]**	
Al(4)	+ 1.58	+ 2.16
Al(5)/Al(6)	+ 0.99/ + 1.11	+ 1.01/ + 1.15
O(3)/O`(3)	−0.68/−0.75	−0.92/−0.89
Ni^1st^	–	+ 0.35
**Bond**	**Bond Order**	
O(3)–Al(5) O(3)–Al(6) O(3)–Ni	0.73/0.66 0.62 –	0.66/0.51 0.53 0.15 (2.09Å)
O’(3)–Al(5) O’(3)–Al(4) O’(3)–Ni	0.66/0.66 0.63 –	0.57/0.55 0.38 0.004 (3.10Å)

**Table 2.** Theoretical DRIFT spectra–characteristic vibrations of water at TiO_2_ and Al_2_O_3_. Data taken from our DFT calculations.

**TiO_2_**	**H_2_O_mol_**	**H_2_O_diss_**	**H_2_O_mol_**	**H_2_O_diss_**
**Vibrational Mode**	**TiO_2_**		**Al_2_O_3_**	
δ(H-O-H)	1,646	–	1,595	–
ν(O(1)H)	3,591	3,623	3,359	3,338
ν(O(2)H)	3,744	3,654	3,692	3,664

**Table 3.** Comparison of vibrational frequencies obtained from theoretical (DFT) codes: StoBe and Gaussian98 with experimental and literature data for pure HNCO.

	**DFT**			***Experiment***	Theory-MP2*
	**StoBe (PBE)**		**Gaussian98 (B3LYP)**		
**Frequencies [cm^−1^]**	**har.**	**unhar.**	**har.**		**har.**
ν(NH)	3,578	3,536	3,678	***3,511***	3,791
ν_as_(NCO)	2,259	2,259	2,356	***2,259***	2,366
ν_s_(NCO)	1,269	1,267	1,338	***1,316***	1,310
δ(HNC)	833	833	780	***770***	794
π(NCO)	583	583	611	***697***	610
δ(NCO)	547	547	562	***573***	559

* Raunier *et al*. [31]; har.–harmonic approximation; unhar.–unharmonic approximation.

**Table 4.** The HNCO adsorption at TiO_2_(101) and γ-Al_2_O_3_ (100): theoretical vibrational frequencies (cm^−1^) of individual surface intermediates.

	**TiO_2_**		**Al_2_O_3_**	
**ν (NCO)**	**Sym.**	**Asym.**	**Sym.**	**Asym.**
HNCO	1,279	2,266	1,263	2,264
NCO_aq_	1,351	2,234	1,342	2,274
NCO	1,345	2,181	1,364	2,293
**ν (N-H/H-N-H)**	**Sym.**	**Asym.**	**Sym.**	**Asym.**
NHCOOH	3,462	−	3,280	−
NH_2_CO_2_	3,346	3,482	3,364	3,488
NH_2aq_	2,905/3,349	3,466	3,406/3,425	3,533
NH_3aq_	2,955/3,381	3,492	3,068/3,394	3,484

**Table 5.** DFT-obtained atomic charges, distances (Å) and bond orders (Mayer bond analysis) of the pure and Ca-doped V_6_O_20_H_10_ cluster modelling the V_2_O_5_ (010) surface.

**Cluster:**		**V_6_O_20_H_10_**		**V_6_O_20_H_10_ + Ca**	
*Centre*		*Charge*		*Charge*	
V(5)		+ 1.39		+ 1.42	
O(1)		−0.33		−0.62	
O(2)		−0.67		−0.65	
O(3)		−0.87		−0.87	
Ca		–		+ 1.10	
*Bond*	*Distance [Å]*	*Bond Order*		*Bond Order*	
O(1)–V(5) O(1)–K	1.57 → 1.62 2.74	2.05 –	∑ = 2.05	1.54 0.30	∑ = 1.84
O(2)–V(5) O(2)–V(5) O(2)–K	1.78 1.78 3.99	0.85 0.85 −	∑ = 1.70	0.87 0.87 0.06	∑ = 1.80
O(3)–V(5) O(3)–V(5) O(3)–V(5) O(3)–K	1.88 1.88 2.01 4.11	0.49 0.49 0.42 −	∑ = 1.40	0.52 0.52 0.39 0.03	∑ = 1.46
*E_HOMO/LUMO_ [eV]*		−6.64/−4.59		−4.06/−4.03	
		***DFT-based****XPS Ionization potentials [eV]*			
O(1)		529.3		530.8	
O(2)		528.9		530.8	
O(3)		529.0		531.0	
O(1)–H		533.5		–	
		***Experimental****XPS Ionization potentials [eV]*			
TiO_2_–WO_3_		529.8/531.7			
hydroxyl groups		533.0			
V_2_O_5_		529.1		531.5	
